# Supplementary material for: Comparative Interaction Studies of Quercetin with 2-Hydroxyl-propyl-β-cyclodextrin and 2,6-Methylated-β-cyclodextrin
Source: Molecules. 2022 Aug 26;27(17):5490. doi: 10.3390/molecules27175490 (PMC9458201; doi:10.3390/molecules27175490)
Supplement: Supplementary file 1 [file molecules-27-05490-s001.zip › molecules-1829167-supplementary.pdf]

Supplementary Materials

Comparative interaction studies of quercetin with 2-hydroxyl-propyl-cycodextrin and 2,6-methylated-cyclodextrin

Vasiliki Vakali[1], Michail Papadourakis[2], Nikitas Georgiou[1], Nikoletta Zoupanou[1], Dimitrios A. Diamantis[3], Uros Javornik[4], Paraskevi Papakyriakopoulou[5], Janez Plavec[4], Georgia Valsami[5*], Andreas G. Tzakos[3,6*], Demeter Tzeli[7,8*], Zoe Cournia[2*] and Thomas Mauromoustakos[1*]

[1] Organic Chemistry Laboratory, Department of Chemistry, National and Kapodistrian University of Athens, Panepistimiopollis Zografou, 11571 Athens, Greece
[2] Biomedical Research Foundation Academy of Athens, 4 Soranou Ephessiou, 11527 Athens, Greece
[3] Department of Chemistry, Section of Organic Chemistry and Biochemistry, University of Ioannina, 45110 Ioannina, Greece
[4] Slovenian NMR Centre, National Institute of Chemistry, SI-1001 Ljubljana, Slovenia
[5] Department of Pharmacy, School of Health Sciences, National and Kapodistrian University of Athens, 15784 Athens, Greece
[6] Institute of Materials Science and Computing, University Research Center of Ioannina (URCI), 45110 Ioannina, Greece
[7] Laboratory of Physical Chemistry, Department of Chemistry, National and Kapodistrian University of Athens, Panepistimioupolis Zografou, 11571 Athens, Greece
[8] Theoretical and Physical Chemistry Institute, National Hellenic Research Foundation, 11635 Athens, Greece
* Correspondence: valsami@pharm.uoa.gr (G.V.); atzakos@uoi.gr (A.G.T.); tzeli@chem.uoa.gr (D.T.); zcournia@bioacademy.gr (Z.C.); tmavrom@chem.uoa.gr (T.M.)

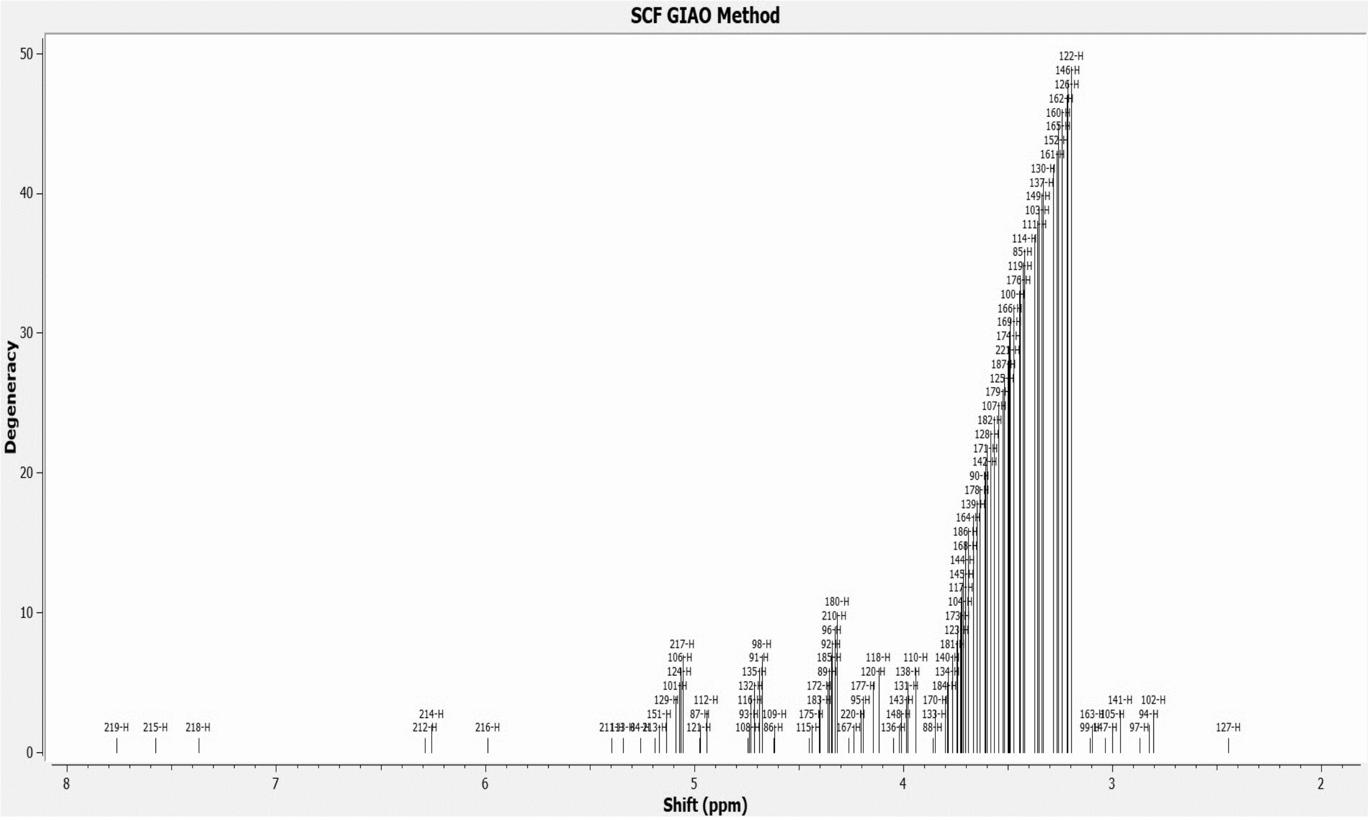

Figure S1: ^{1}H NMR chemical shifts for the 2,6Me-β-CD-QUE complex, with SCF GIAO method.

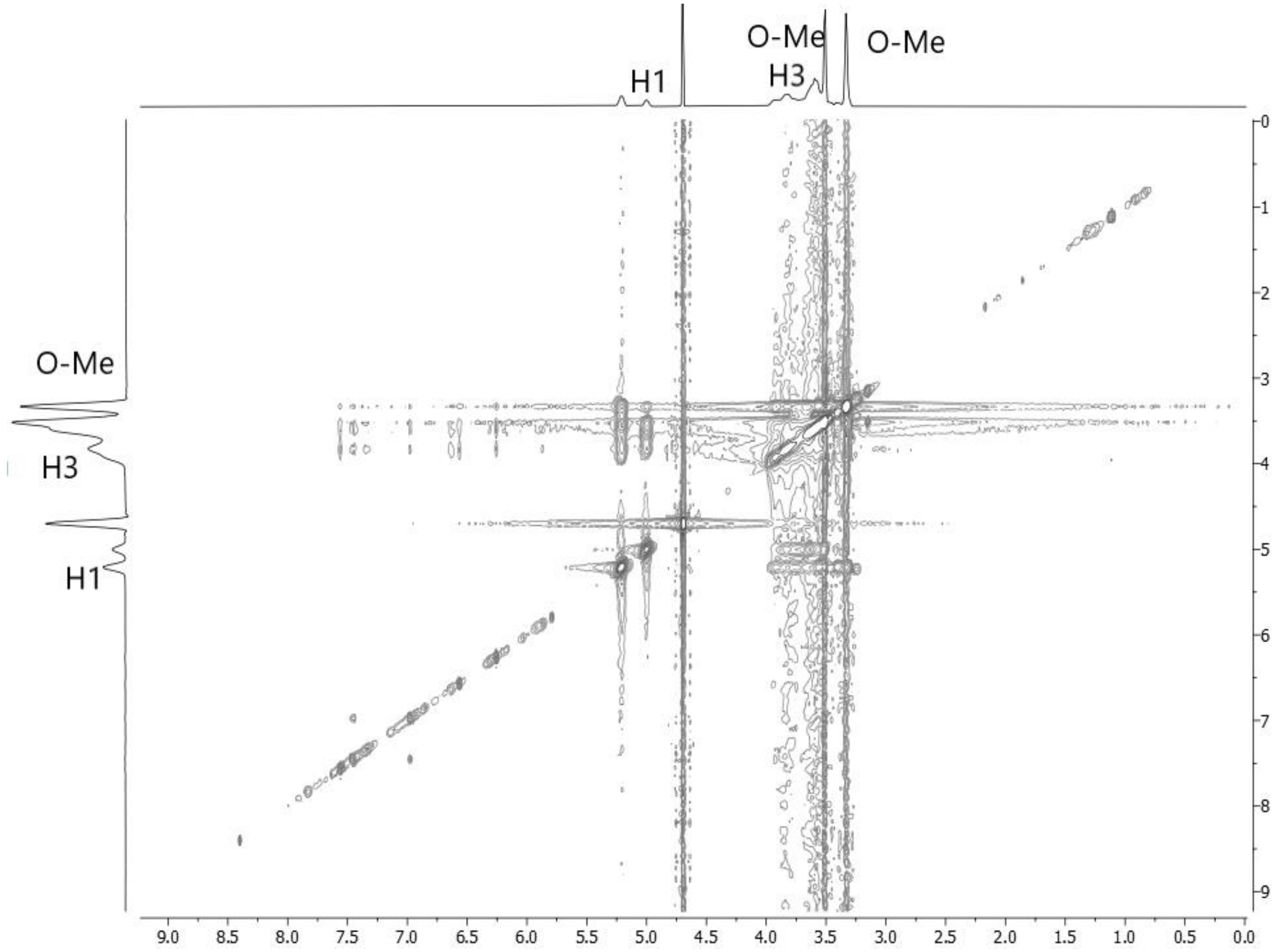

Figure S2: 2D NOESY spectrum obtained in a 400 MHz Bruker spectrometer of QUE-2,6Me-β-CD complex at 25 ° C in D_2O.

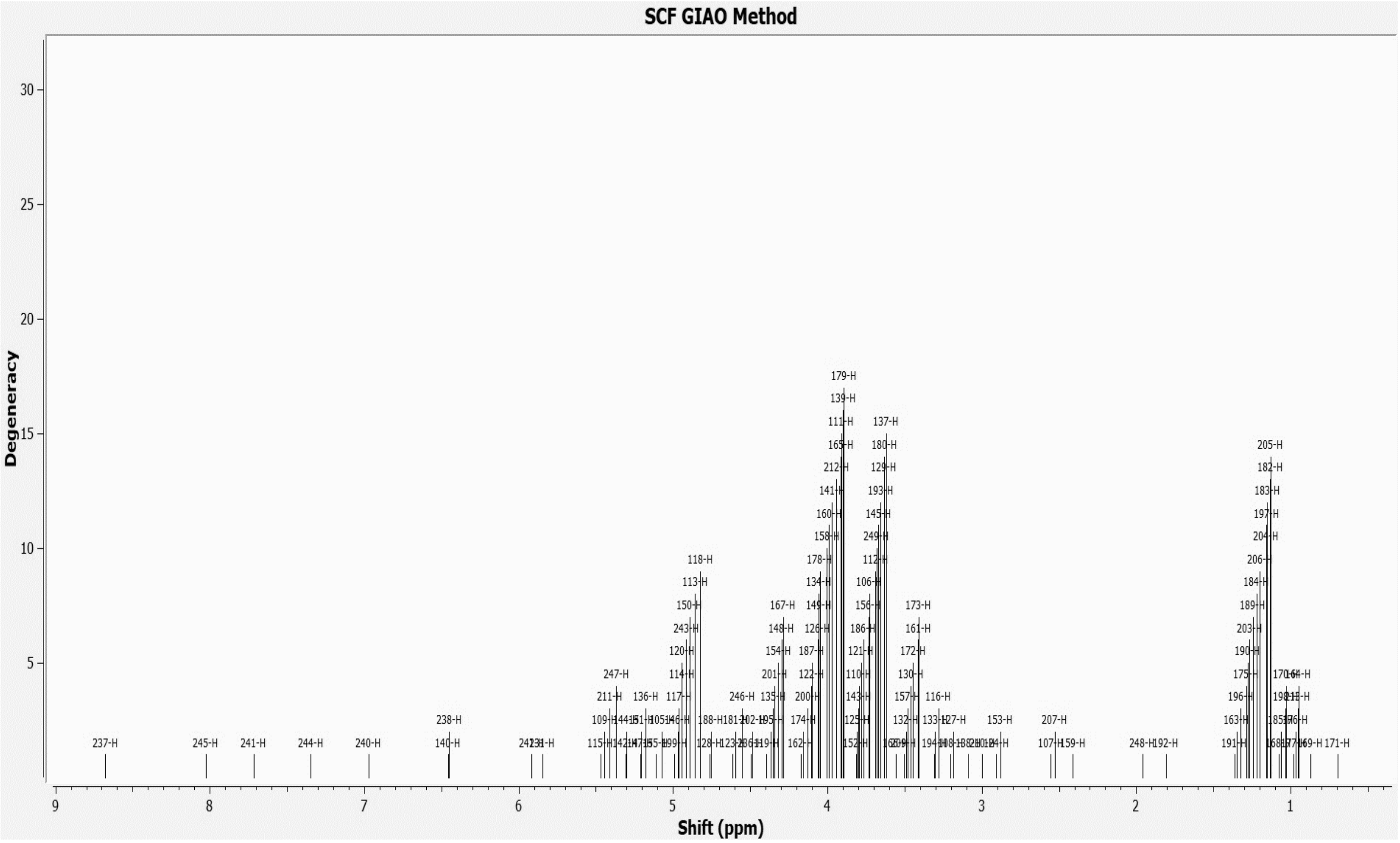

Figure S3: ^{1}H NMR chemical shifts for the QUE-2HP-β-CD complex, with SCF GIAO method.

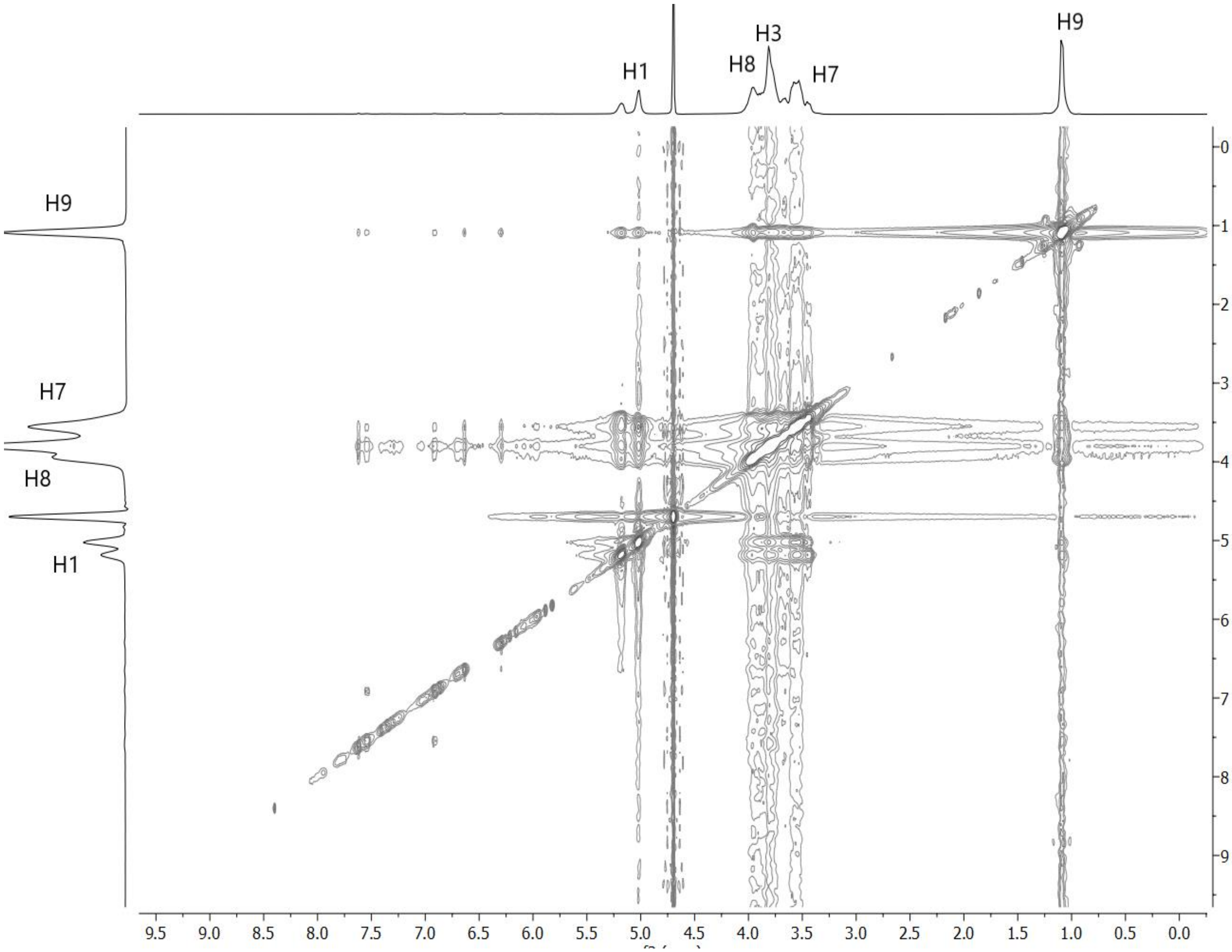

Figure S4: 2D NOESY spectrum obtained in a 400 MHz Bruker spectrometer of QUE-2HP-β-CD complex at 25 ° C in D_2O.

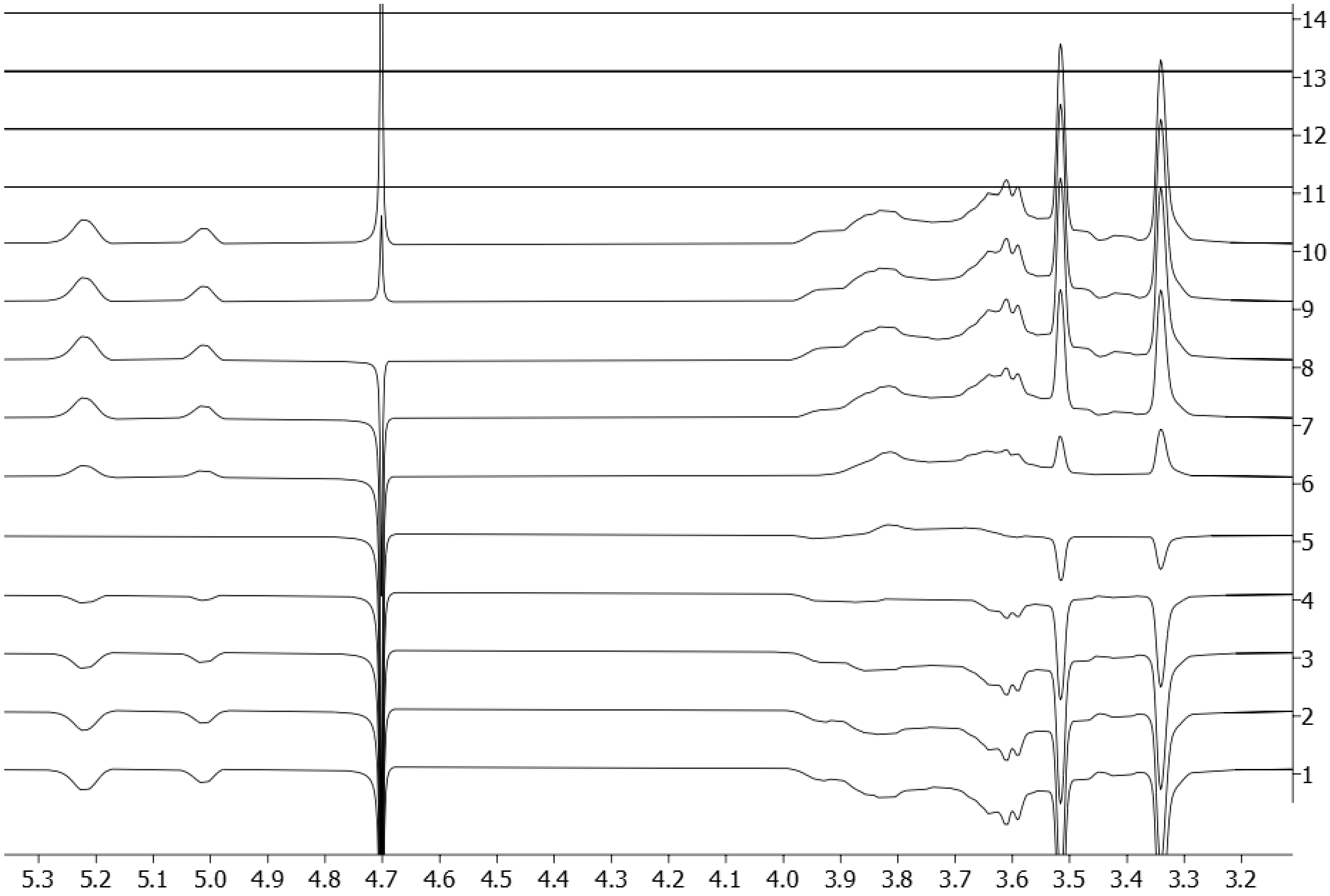

Figure S5: T_1 ^{1}H-NMR series of experiments for the 2,6Me-β-CD. The interval time between the two pulses (t) from bottom to top expressed in seconds are: 0.01s, 0.05s, 0.10s, 0.25s, 0.50s, 1.00s, 2.00s, 4.00s, 8,00s, 15,00s.

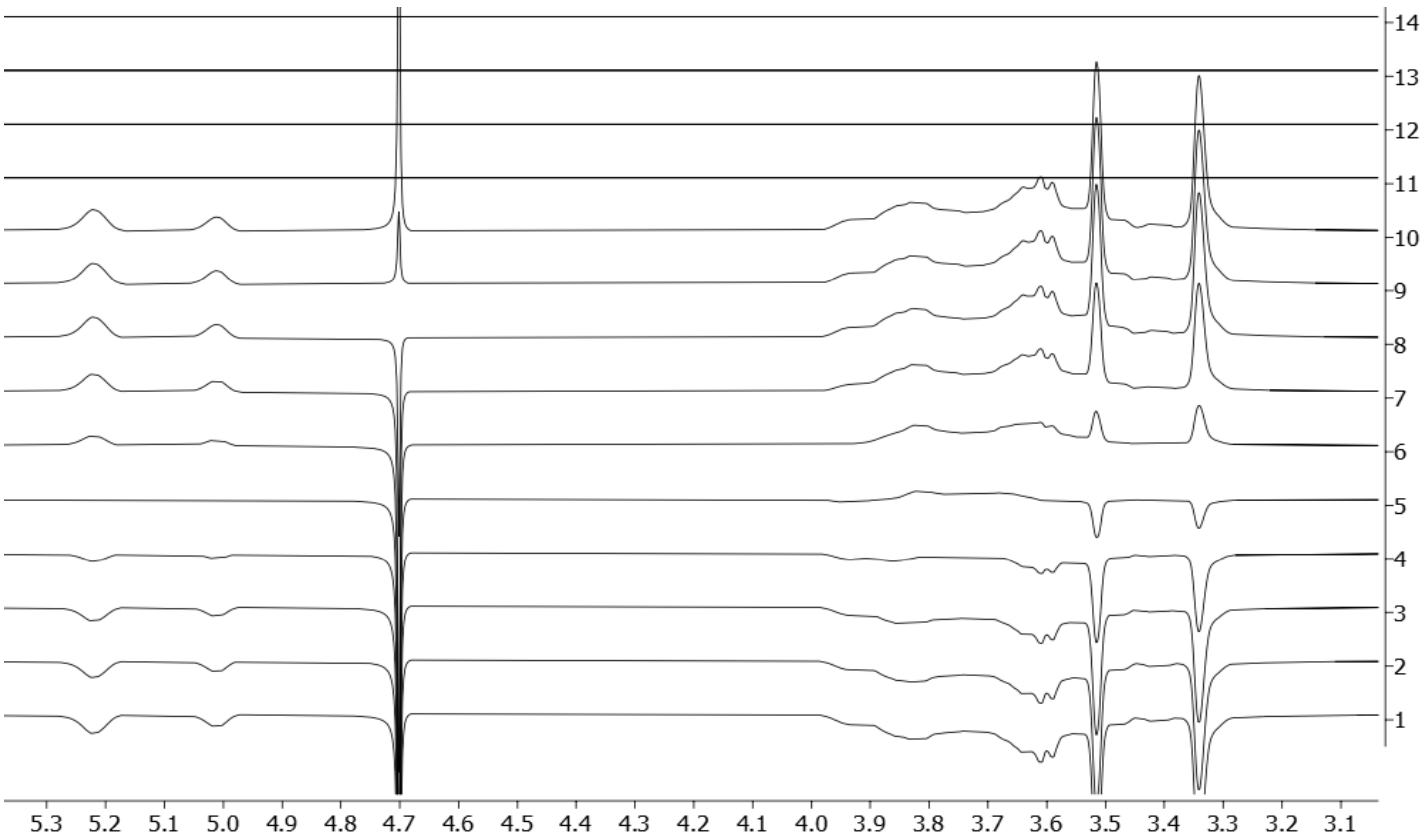

Figure S6: T_1 ^{1}H-NMR series of experiments for the QUE-2,6Me-β-CD complex. The interval time between the two pulses (t) from bottom to top expressed in seconds are: 0.01s, 0.05s, 0.10s, 0.25s, 0.50s, 1.00s, 2.00s, 4.00s, 8,00s, 15,00s.

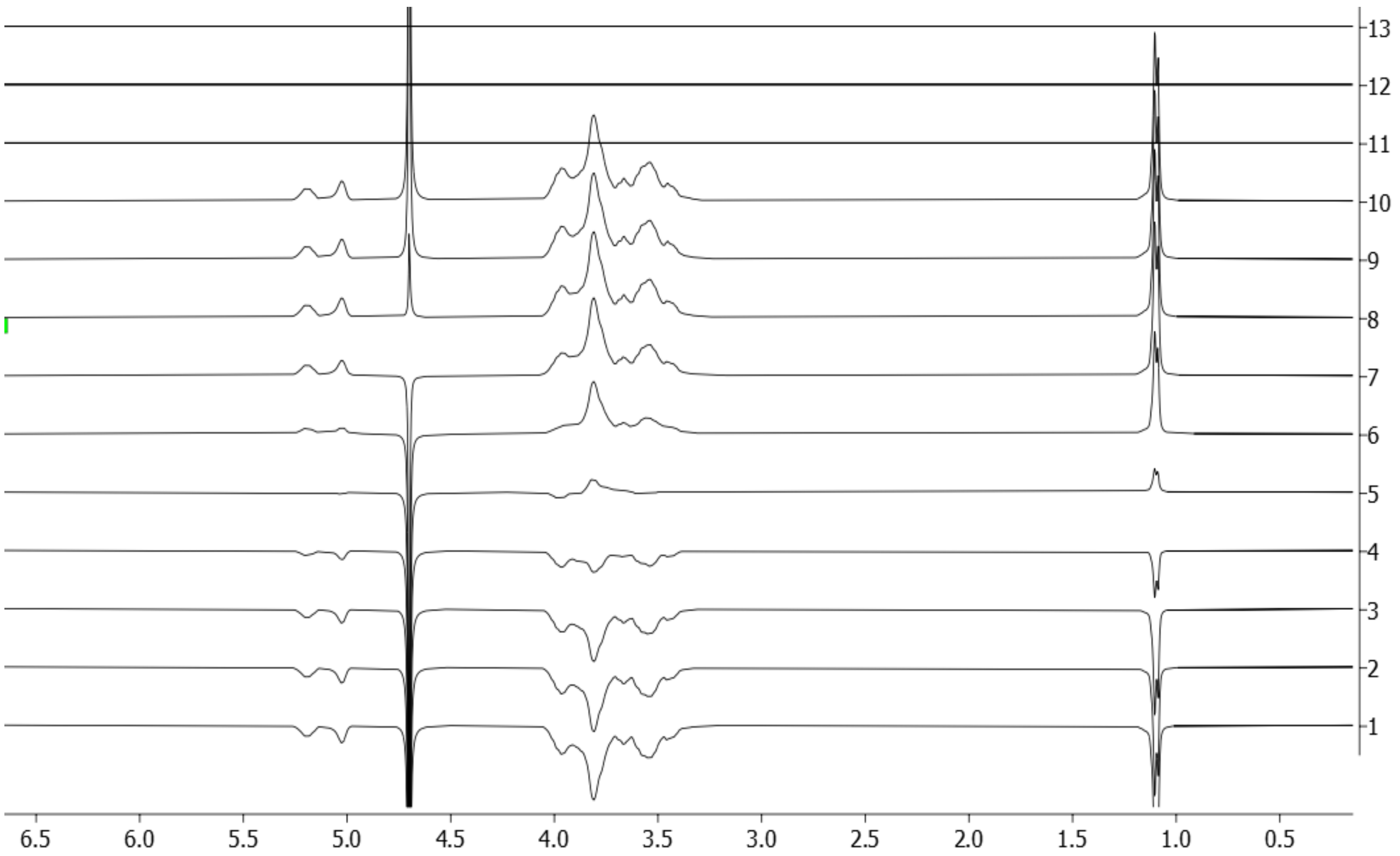

Figure S7: T_1 ^{1}H-NMR series of experiments for the 2HP-β-CD. The interval time between the two pulses (t) from bottom to top expressed in seconds are: 0.01s, 0.05s, 0.10s, 0.25s, 0.50s, 1.00s, 2.00s, 4.00s, 8,00s, 15,00s.

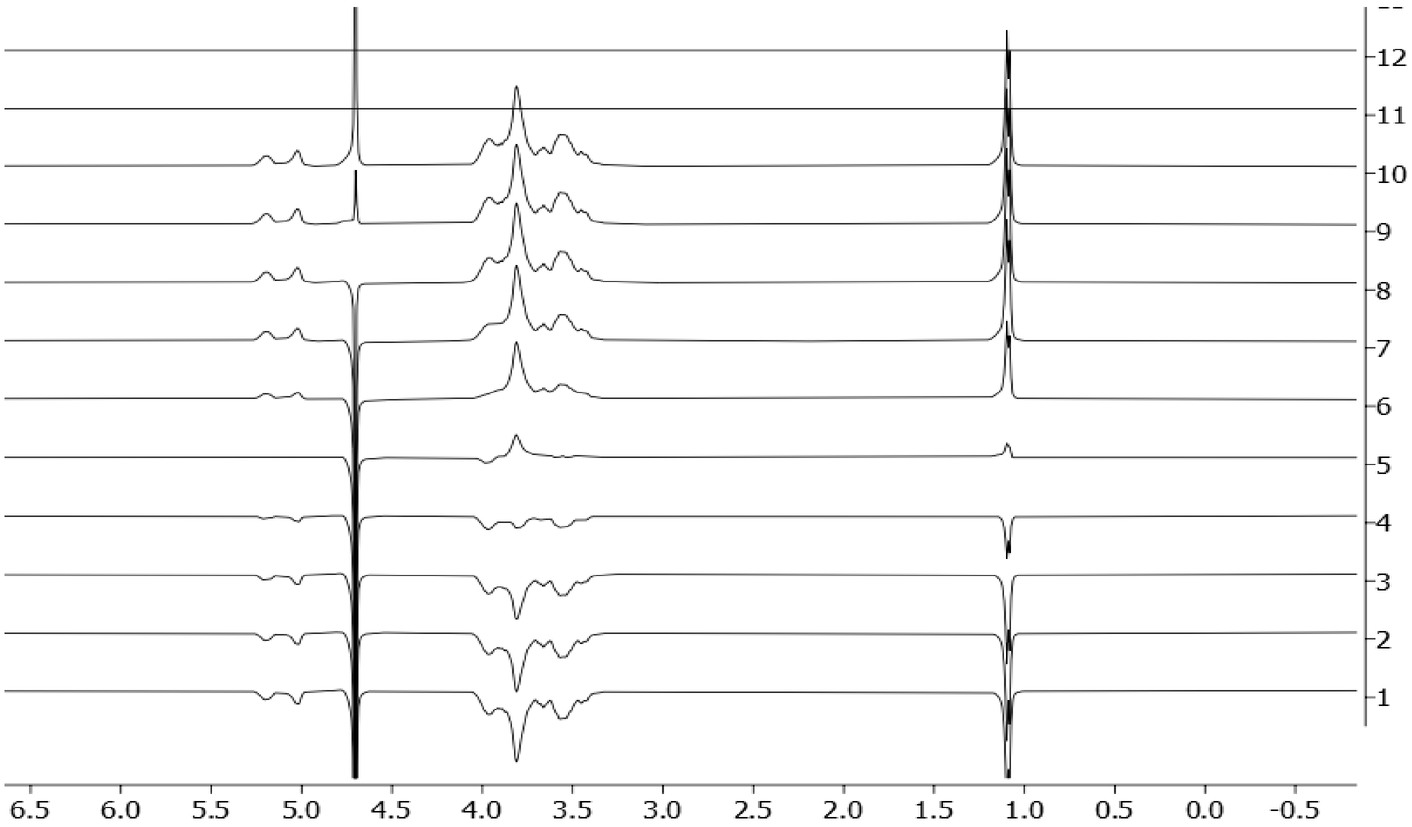

Figure S8: T_1 ^{1}H-NMR series of experiments for the QUE-2HP-β-CD complex. The times between the two pulses (t) from bottom to top expressed in seconds are: 0.01s, 0.05s, 0.10s, 0.25s, 0.50s, 1.00s, 2.00s, 4.00s, 8,00s, 15,00s.

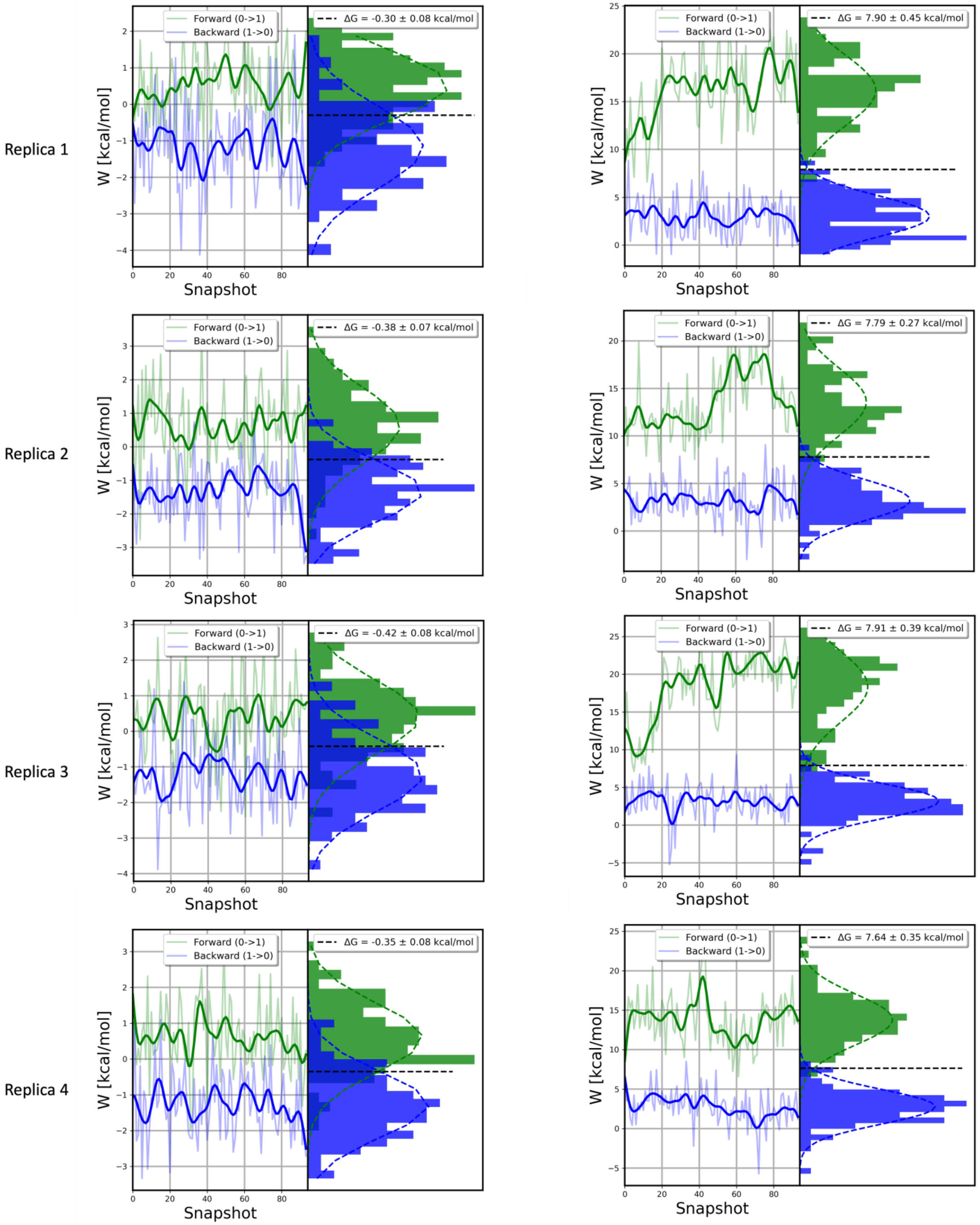

Figure S9: Work values and the distribution of the work values from the QUE solvation leg of the thermodynamic cycle (on the left) and for the QUE-2HP-β-CD complex, and the QUE-2HP-β-CD coupling leg of the thermodynamic cycle (on the right) forward (green) and reverse (blue) directions. The transition length for each transition was 500 ps, and the work values depicted here are measured for each transition.

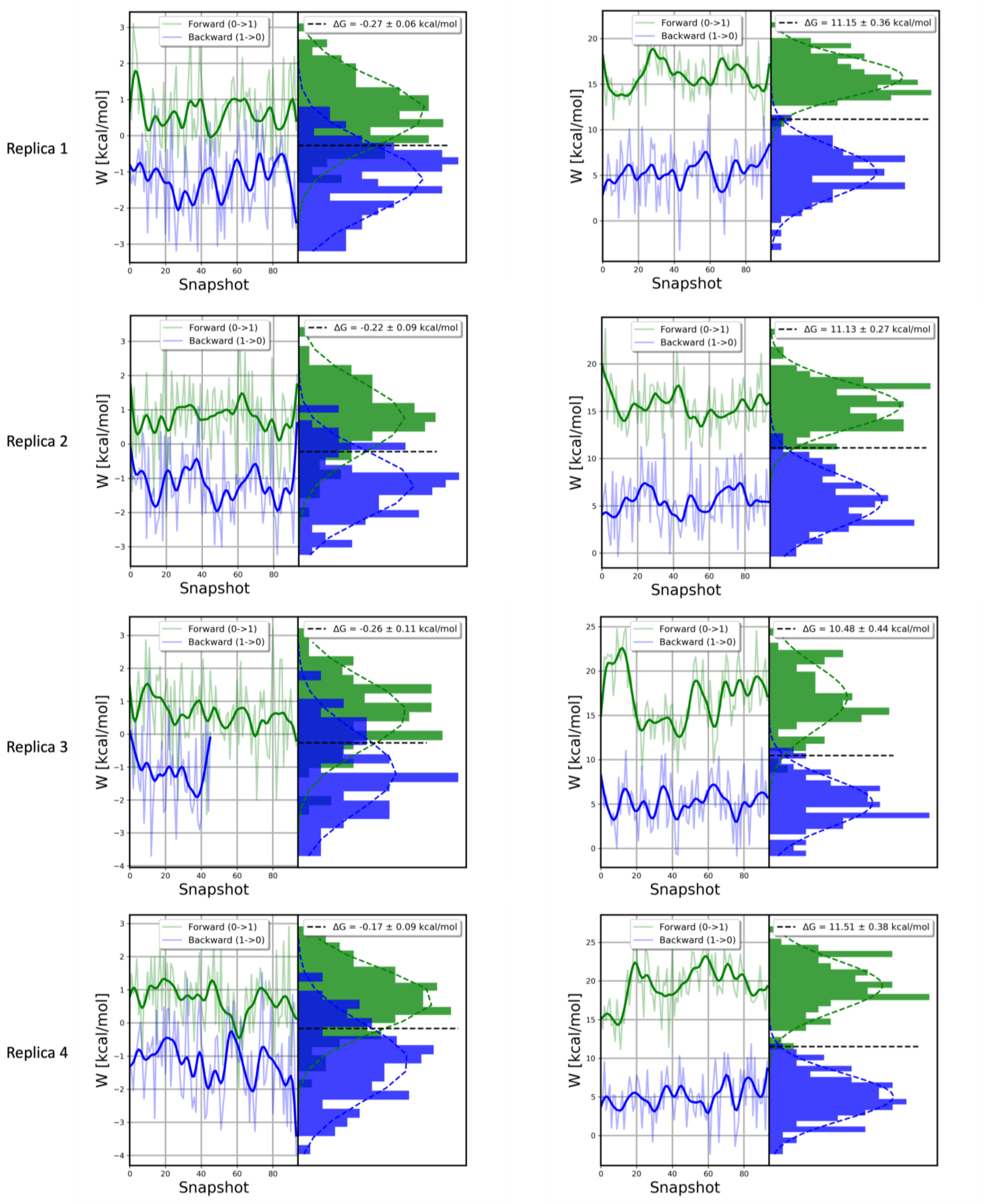

Figure S10: Work values and the distribution of the work values from the QUE solvation leg of the thermodynamic cycle (on the left) and for the QUE-2HP-β-CD complex, and the QUE-2,6Me-β-CD coupling leg of the thermodynamic cycle (on the right) forward (green) and reverse (blue) directions. The transition length for each transition was 500 ps, and the work values depicted here are measured for each transition.
